# Native microbiome dominates over host factors in shaping the probiotic genetic evolution in the gut

**DOI:** 10.1038/s41522-023-00447-8

**Published:** 2023-10-14

**Authors:** Shuaiming Jiang, Chengcheng Zhang, Zhe Han, Wenyao Ma, Shunhe Wang, Dongxue Huo, Weipeng Cui, Qixiao Zhai, Shi Huang, Jiachao Zhang

**Affiliations:** 1 Key Laboratory of Food Nutrition and Functional Food of Hainan Province, School of Food Science and Engineering, Hainan University, Haikou, 570228 China; 2 School of Food Science and Technology, Jiangnan University, Wuxi, Jiangsu China; 3 Faculty of Dentistry, The University of Hong Kong, Hong Kong SAR, China; 4 One Health Institute, Hainan University, Haikou, Hainan 570228 China

**Keywords:** Microbial genetics, Bacteria

## Abstract

Probiotics often acquire potentially adaptive mutations in vivo, gaining new functional traits through gut selection. While both the host and microbiome can contribute to probiotics’ genetic evolution, separating the microbiome and the host’s contribution to such selective pressures remains challenging. Here, we introduced germ-free (GF) and specific pathogen-free (SPF) mouse models to track how probiotic strains, i.e., *Lactiplantibacillus plantarum* HNU082 (Lp082) and *Bifidobacterium animalis* subsp. *lactis* V9 (BV9), genetically evolved under selection pressures derived from host factors alone and both host and microbial ecological factors. Notably, compared to the genome of a probiotic strain before consumption, the host only elicited <15 probiotic mutations in probiotic genomes that emerged in the luminal environment of GF mice, while a total of 840 mutations in Lp082 mutants and 21,579 mutations in BV9 were found in SPF mice, <0.25% of those derived from both factors that were never captured by other experimental evolution studies, indicating that keen microbial competitions exhibited the predominant evolutionary force in shaping probiotic genetic composition (>99.75%). For a given probiotic, functional genes occurring in potentially adaptive mutations induced by hosts (GF mice) were all shared with those found in mutants of SPF mice. Collectively, the native microbiome consistently drove a more rapid and divergent genetic evolution of probiotic strains in seven days of colonization than host factors did. Our study further laid a theoretical foundation for genetically engineering probiotics for better gut adaptation through in vitro artificial gut ecosystems without the selection pressures derived from host factors.

## Introduction

Probiotics face tremendous challenges in colonizing and adapting to the luminal environment of the host gut. The main barriers include (i) specific characteristics of a probiotic itself (e.g., acid tolerance, the frequency and dosage of intake, live or dead, and specialized adaptability); (ii) host-related factors (such as stomach acidity, bile acids, defensins, immune response, etc.)^1^, (iii) microbiome-related factors (such as microbial resource competition and interactions)^2,3^. All these constraints together constitute intestinal selection pressures on exogenous microbes in vivo^4^. Such selection pressures persistently drive probiotics to mutate themselves and compete with other residents for limited ecological niches to better survive in the host gut during colonization, leading to many unseen new phenotypes and raising the chance of becoming specialists in the new environment.

Consequently, probiotic adaptive evolution is of great interest in downstream applications, as it often leads to enhanced varied therapeutic efficacy and produces well-gut-adapted strains with superior probiotic characteristics in feces^5^. More importantly, it also implies the possibilities of genetically engineering probiotics with an ecological approach. Firstly, under in vitro conditions, most probiotics are genetically stable. For example, in our past study, the candidate probiotic *Lactiplantibacillus plantarum* HNU082 (Lp082) did not accumulate any mutations throughout the continuous in vitro incubation over 28 days. In another study, along the 2000-generation in vitro cultivation for ten months under antibiotic exposure, only less than 20 mutations were observed^6^. In contrast, microbial adaption to the gut requires ingested probiotics to acquire new phenotypes via a high number of adaptive genetic mutations. Our previous study found that probiotic *Lactiplantibacillus plantarum* HNU082 (Lp082) acquired the potentially adaptive mutations conservatively in the host gut of mice and humans, enhancing its acid tolerance and rhamnose utilization within a short colonization duration^7^. Both host-derived factors and resident gut microbiota can impose selection pressure on probiotics and lead to these potentially adaptive mutations. Yet, tracing which factor (i.e., host-derived factors VS. resident gut microbiota) contributed more to probiotic genetic evolution remains challenging.

On the other hand, our previous studies on in vivo adaptive evolution also motivated us to genetically engineer probiotics using ecological forces in gut ecosystems. The direct gut passage of probiotics using animals (e.g., mice) was effective, yet not optimal for the large-scale and efficient production of probiotic mutants. Alternatively, an artificial gut ecosystem teeming with designed competitors of target probiotic strains seems ideal, in which no host-factor-related selection pressure will appear. However, it remains elusive how large the relative host contribution to the probiotic mutations would be, which would be critical to selecting the artificial gut ecosystem for such a purpose.

**Fig. 1:**
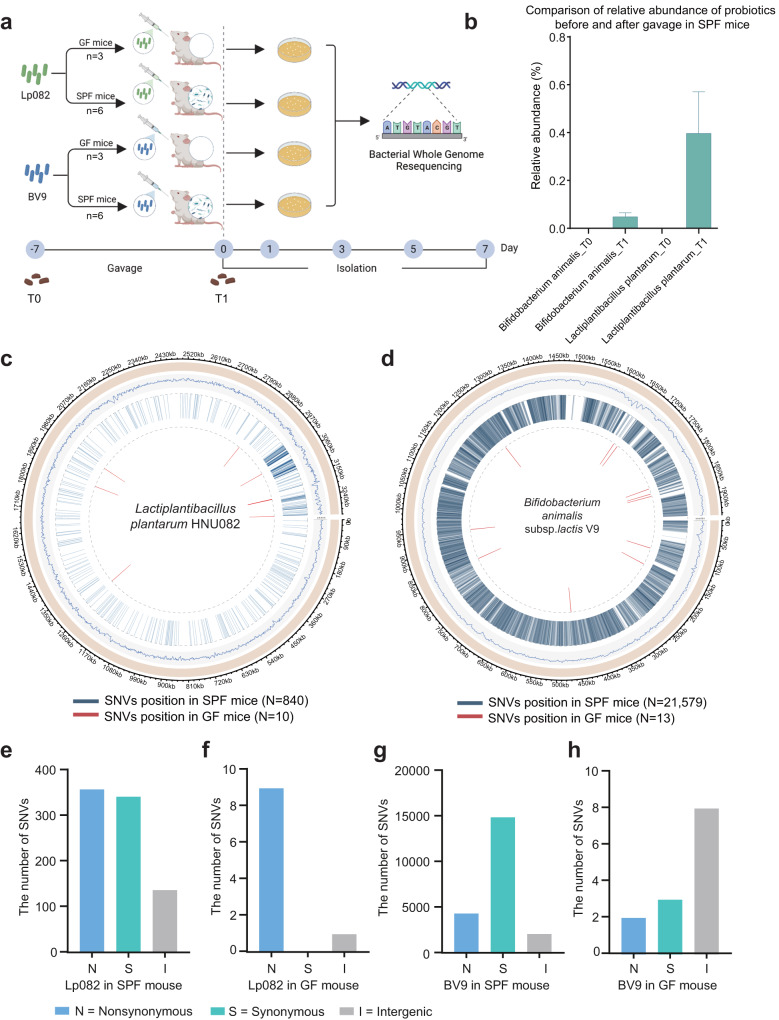
The potentially adaptive mutations of two probiotic strains in GF and SPF mice, respectively. **a** Experimental design. GF and SPF mice were recruited to administer probiotics (10^8^ cfu/day) for seven days, and the fecal isolates were obtained on the following 1, 3, 5, and 7 days. Feces were collected before and after gavage for metagenomic sequencing. **b** The relative abundance of probiotics before and after gavage in SPF mice was compared and was represented by a bar chart with error bars (Standard Deviation, SD). Before gavage, there were no probiotics and similar species in the resident microbiota. **c**, **d** The mutation sites that occur in all isolates were shown. The circle map shows the genome-wide distribution of point mutation in the ingested probiotic strains in SPF and GF mice, respectively. A blue stripe indicates the probiotic’s SNVs position in SPF mice, while a red stripe represents that accordingly in GF mice. **e**, **f** For Lp082, a total of 702 mutations were located in gene-coding regions, with 138 mutations occurring in non-coding regions in SPF mice. In GF mice, a total of 9 mutations were located in gene-coding regions, with 1 mutation occurring in a non-coding region. **g**, **h** For BV9, a total of 19,396 mutations were located in gene-coding regions, with 2183 mutations occurring in non-coding regions in SPF mice. In GF mice, a total of 5 mutations were located in gene-coding regions, with 8 mutations occurring in non-coding regions.

Since *Lactiplantibacillus* and *Bifidobacterium* were two major groups of administrated probiotics^8^, here, we employed Lp082 and *Bifidobacterium animalis* subsp. *lactis* V9 (BV9) as the model probiotic strains to quantitively measure the contribution of the host and microbial factors to the in vivo genetic evolution of probiotics^7,9^. Lp082, a representative strain of *Lactiplantibacillus plantarum* derived from fermentation foods, can acquire potentially adaptive genetic mutations during a short-term gut passage in zebrafish, mice, and humans^7^. Notably, these potentially adaptive mutations or involved functional genes were highly consistent across individuals of a host species and even across host species. On the other hand, BV9, which originated from the feces of a healthy Mongolian child in China, is a representative *Bifidobacteria* strain that has been commercialized in the Chinese market^10^ with promising probiotic functions^11^. Although the experimental evolution study is still lacking for this strain, it is widely accepted that *Bifidobacteria* often harbors a high strain-level diversity in the human gut and a large genomic flexibility for adaption to ecological challenges. This suggests that BV9 may have a similarly high potential for adaptive evolution in the gut environment. The probiotic strains (10^8^ cfu/day) were administrated to germ-free (GF) and specific pathogen-free (SPF) mice over seven days and isolated and cultivated from the feces every two days for genomic analysis (Fig. 1a). The identity of both strains was verified with strain-specific antibiotics and DNA primers^7^. Next, the whole-genome sequence of isolates was obtained and mapped against that of the original strains to gain potentially adaptive SNVs (Single nucleotide variants). The SNVs detected in GF and SPF mice were then analyzed and compared, providing quantitative insights into the host and microbiome contribution to adaptive genetic changes of probiotics in the host gut. This study also laid the theoretical foundation for developing a directed evolution strategy of probiotics using in vitro artificial gut ecosystems.

## Results

### Native microbiota was a major force driving the potentially adaptive mutations

To gain insights into the selective forces for ingested probiotic strains in the host gut, we first pinpoint the model probiotic strains to perform the experimental evolutionary study.

We first profiled the composition of fecal microbial communities of SPF mice using MetaPhlAn3 and confirmed that *Bifidobacterium animalis* and *Lactiplantibacillus plantarum* were not present in the gut of healthy SPF mice before probiotics gavage. It is also noteworthy that both probiotic species were able to be annotated in the gut microbiome of mice after gavage, reaching a meaningful abundance level (Fig. 1b), suggesting that the probiotics were able to successfully survive and potentially exert their beneficial effects on the host. To be noted, we demonstrated that the genetic mutations of probiotics were only attributed to the probiotic strains we introduced rather than any pre-existing probiotic strains in the gut of mice.

While only ten mutations occurred in Lp082 passing the GI tract of GF mice, it acquired one order of magnitude more potentially adaptive mutations (*N* = 840) in SPF mice. The host contribution to potentially adaptive mutations in Lp082 was only 0.24%, whereas the microbiome contribution reached 99.76% (Fig. 1c). Likewise, for BV9, only 13 mutations were identified from GF mice, which were supposed to arise purely from host factors. A total of 21,579 mutations were found in isolates from SPF mice; thus, the host contribution was only 0.05% (Fig. 1d). These suggested that: (i) the core selection pressure for ingested probiotics primarily came from resource competitions between microbes rather than host factors; (ii) *Bifidobacterium* more actively mutated than lactic acid-producing bacteria by two orders of magnitude^12^. For SPF mice, Lp082 had a total of 359 mutations involving amino acid substitutions (Fig. 1e), defined as nonsynonymous mutations, and for BV9, there were 4428 nonsynonymous mutation sites (Fig. 1f). In GF mice, Lp082 had 9 SNVs involving amino acid substitutions, and BV9 had 2 nonsynonymous mutation sites (Fig. 1g, h).

### Divergent evolution of probiotics during colonization in the gut of SPF mice

**Fig. 2:**
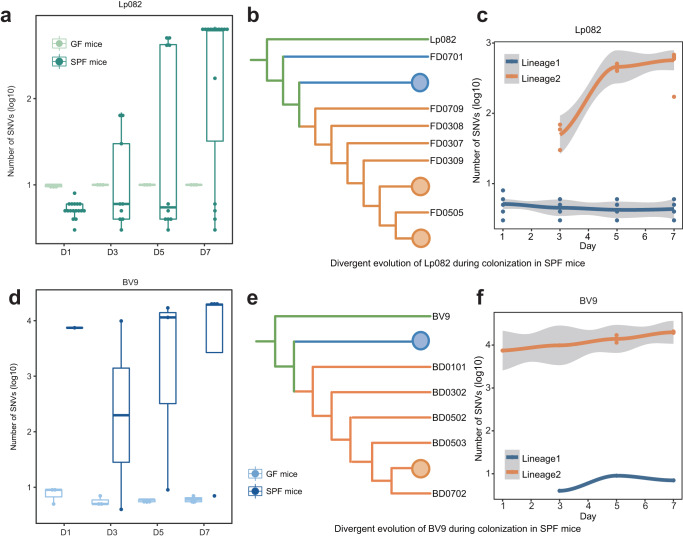
Divergent evolution of probiotics in vivo. **a** The number of SNVs of Lp082 during colonization in GF and SPF mice. **d** The number of SNVs of BV9 during colonization in GF and SPF mice. **b**, **e** Based on the number of SNVs of isolates, the phylogenetic tree reorganized all isolates of Lp082 and BV9, respectively. Different colors in the branches show divergent lineages. **c**, **f** Two lineages were observed in Lp082 and BV9 separately. The number of SNVs in divergent lineages was plotted against time, where its temporal patterns were highlighted with the fitted curves.

During this longitudinal study, both strains mutated slightly and maintained a low yet stable level over time in GF mice. However, in SPF mice, the number of potentially adaptive mutations gradually increased during colonization, with a large variation observed across isolates (Fig. 2a, d). Based on the large variation of SNVs, we found that both the isolates of Lp082 and BV9 experienced divergent selection and rapidly evolved into two divergent lineages in the gut of SPF mice (Fig. 2b, e). Intriguingly, strains from one lineage carried fewer mutations and maintained a conservative evolutionary trend, while the other lineage carried enormous mutations (Fig. 2c, f). Here we first demonstrated and compared in vivo divergent evolutionary patterns of representative probiotic strains.

### Conserved functional genes in potentially adaptive mutations of probiotics residing in both GF and SPF mice

**Fig. 3:**
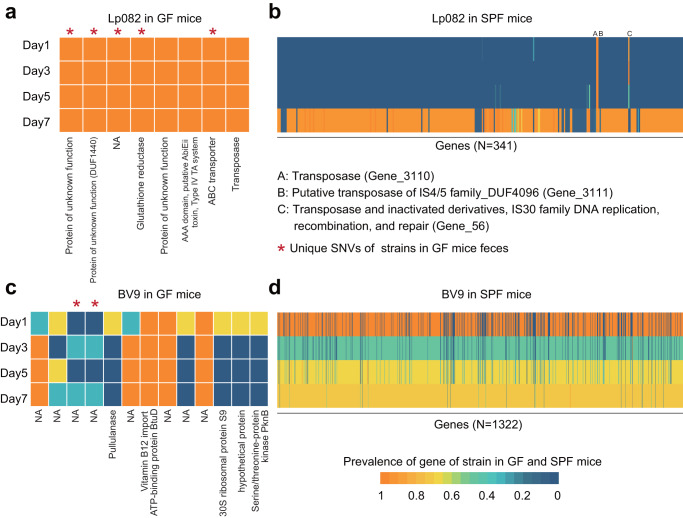
The functional annotation of mutated genes of Lp082 and BV9. **a**, **c** The functional annotation of mutated genes of Lp082 and BV9 in GF mice feces. The asterisk indicates the unique SNVs of strains in GF mice feces compared with SNVs of isolated strains in SPF mice. The heatmap color indicates the mutation frequency of a gene in the sample at different time points. **b**, **d** The prevalence of mutated genes of Lp082 and BV9 in the feces of SPF mice. There were 341 mutated genes in Lp082 and 1322 in BV9 under the bacterial condition.

We next functionally annotated genes involved in these mutations (Fig. 3). The potentially adaptive mutations of Lp082 involved seven genes in GF mice and 341 genes in SPF mice (Fig. 3a, b), while the potentially adaptive mutations of BV9 involved five genes in GF mice and 1322 genes in SPF mice (Fig. 3c, d). Although unique SNVs were detected in GF mice, the involved functions all overlapped with those in SPF mice (Supplementary Tables). BV9’s mutations in SPF mice were mainly located on genes responsible for carbohydrate metabolism, e.g., genes encoding beta-galactosidase (Gene_456), bifunctional beta-D-glucosidase/beta-D-fucosidase (Gene_142) and the gene encoding aldehyde-alcohol dehydrogenase (Gene_359). In contrast, the high-frequency mutation sites of Lp082 were mainly related to transposase and inactivated derivatives.

### Intestinal selective pressure introduced by complex species’ interaction drives more genome mutations

**Fig. 4:**
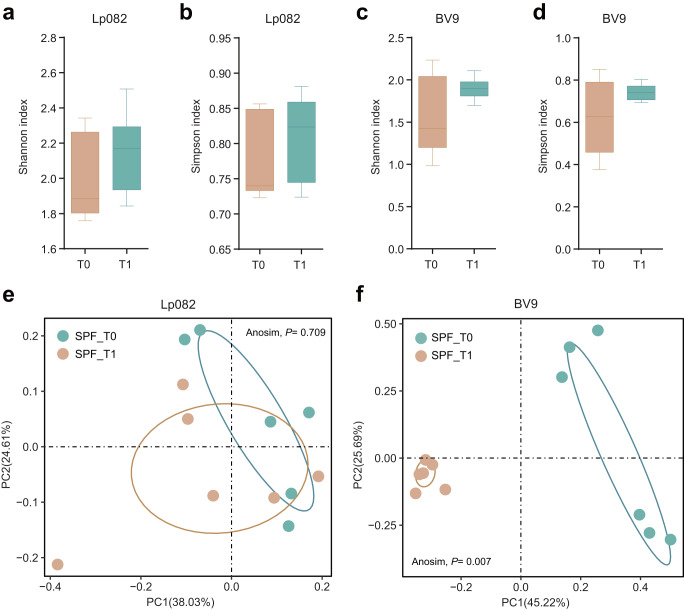
The diversity and structural characteristics of the bacterial microbiota before and after gavage. **a**, **b** The microbial alpha diversity of microbiota before and after Lp082 administration was compared, including the Shannon and Simpson index. **c**, **d** The microbial alpha diversity of microbiota before and after Lp082 administration was compared, including the Shannon and Simpson index. **e**, **f** The PCoA analysis based on the Bray-Curtis distance was employed to compare the structure before and after probiotics administration. The Anosim (Analysis of similarities) was employed to assess significance, indicating non-significant differences in microbial community structure before and after Lp082 ingestion (Anosim*, P* = 0.709). However, following the BV9 intervention, the microbial community exhibited a significant response (Anosim*, P* = 0.007).

**Fig. 5:**
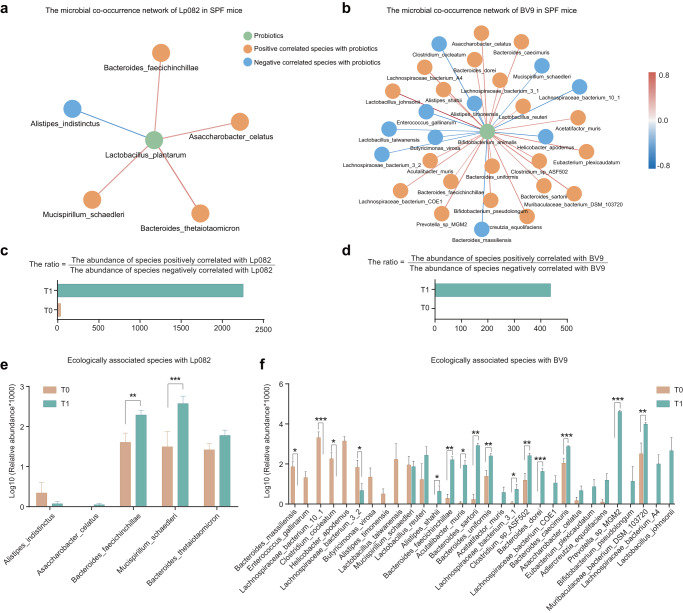
The microbial co-occurrence network based on taxonomic abundance profiles at the species level. **a**, **b** The microbial co-occurrence network of probiotics in SPF mice was constructed based on Spearman analysis with an absolute correlation strength >0.4. The network consisted of green nodes representing the probiotics and orange and blue nodes representing species with a positive and negative correlation with the probiotics. The color of the lines connecting the nodes ranged from blue to orange, indicating the strength and direction of the correlation, with blue representing a negative correlation and orange representing a positive correlation. **c**, **d** The abundance ratio of bacterial species positively correlated with the probiotics divided by those negatively correlated with the probiotics at each time point were compared. **e**, **f** The abundance of species interacting with probiotics before and after probiotic administration was compared and showed with error bars (Standard Deviation, SD). The significance was shown as *P < 0.05, **P < 0.01, and ***P < 0.001 using Wilcoxon signed-rank test.

We next wonder if any native microbiota is associated with the probiotic’s evolution in SPF mice and the impact of the probiotic intervention on the local microbiota. There was no significant difference in the evenness and richness of species composition before and after probiotic administration, as observed through Simpson and Shannon indices (Fig. 4). However, PCoA analysis revealed that the gavage of BV9 had a more prominent impact on the structure of the resident gut microbiota than Lp082 (Fig. 4e, f). Furthermore, the microbial co-occurrence network based on taxonomic abundance profiles at the species level was established, and the correlation between a resident and probiotic strain might suggest their ecological relationships in the gut ecosystem (Fig. 5a, b). As a result, five species (*Bacteroides thetaiotaomicron, Alistipes indistinctus*, etc.) interacted with Lp082, and 33 species (*Bacteroides massiliensis*, *Bacteroides caecimuris*, *Bacteroides uniformis*, etc.) interacted with BV9 with an absolute correlation strength >0.4. To further investigate the effects of the introduced probiotics on the gut microbiota, we computed the abundance ratio of bacterial species positively correlated with the probiotics divided by those negatively correlated with the probiotics at each time point. Interestingly, this ratio increased after introducing the probiotics, indicating that these species positively correlated with the probiotics being substantially enriched. Conversely, those negatively correlated species were depleted during the interaction with the probiotics (Fig. 5c, d).

Accordingly, by comparing the abundance of interacting species before and after probiotic administration, we found that species that had a positive correlation with probiotics showed an increase in abundance after ingestion, while species that had a negative correlation showed a decrease in abundance (Fig. 5e, f). *Mucispirillum schaedleri* showed a positive correlation with Lp082 and a negative correlation with BV9, while *Asaccharobacter celatus* and *Bacteroides faecichinchillae* showed an increase in abundance with both probiotics.

## Discussion

To date, experimental evolution studies on human-associated microbes have mainly been limited to bacterial pathogens due to their profound implications for human health. It was documented that human-associated pathogens evolved various complex virulence factors with limited genetic mutations accumulated to facilitate their immune escape in the host process^13–16^. Probiotics represent another group of human-associated microbes. Due to the gut environment’s ecological nature, probiotics’ genetic changes will undoubtedly occur. However, it is unclear whether the similar evolutionary patterns of pathogens well documented can be found in probiotics and whether host factors such as immunity still primarily shape the evolution of ingested probiotics. Therefore, we carried out experiments to study the adaptive evolution of common probiotics in the gut and try to identify the main driving forces of these genetic mutations.

In contrast, the mutation frequency in probiotics, either *Lactobacillus* or *Bifidobacterium*, far exceeds that of pathogenic bacteria reported to date, which might be due to the distinct ecological properties of probiotics and pathogens. Overall, rapid microbial adaptation within hosts depends on multiple factors, such as the occurrence rate of potentially beneficial mutations, adequate population size, and the fitness advantage of mutants^17^. It is evident that both the mutation rate and effective population size of *M. tuberculosis* in the host were quite small^18–20^, likely due to the remarkably slow growth rate of *M. tuberculosis* within the host. This suggested that *M. tuberculosis* is unlikely to evolve drastically during a single infection within the host. In this study, the strain-level diversity of both probiotic strains increased far more rapidly and drastically within a short timeframe than many other host-associated pathogens, such as *Clostridium difficile*^21,22^ and *Staphylococcus aureus*^23^, which exhibit <10 mutations per genome per year. Thus, we have evidence to believe probiotics might have distinct ecological properties from pathogens in the gut, boosting their potentially adaptive mutations. Firstly, the probiotic population could acquire many beneficial mutations in the genome, as their gut adaptation directly affected the strain’s survival, which was a matter of life and death^24^. In addition, other than host factors, probiotics should resist substantial ecological selection pressures from other residents in the colonization or survival. The well-accepted fact is that strong selection pressure exists between competing species or strains, leading to a negative correlation within co-occurrence networks observed in the metagenomic results^25–27^. Thus, it is possible that the greater number of species with a “direct” negative correlation interacting with BV9 in the gut microbiota may lead to BV9 undergoing a higher number of potentially adaptive mutations than Lp082. The adaptive potential for adaptive mutations of these mutants will be tested in the upcoming research process, which will offer insights into how these genetic changes may contribute to a probiotic’s ability to colonize in the gut environment.

The potential adaptability disparity between lineage-2 and lineage-1 isolates may need to be discussed, and the probiotic bacteria of lineage 2 may evolve well by acquiring more SNVs than lineage 1. Firstly, after the introduction of probiotic bacteria via gavage in SPF mice, the proportion of lineage 2 gradually increased during the isolation process among the mutant strains obtained. This trend demonstrated that lineage 2 displayed a higher degree of suitability within the gut environment compared to lineage 1. Secondly, researches indicated that lineages carrying a higher number of mutations exhibit enhanced adaptability to the host. *Neisseria gonorrhoeae* acquired more genetic factors that enabled it to establish colonization on the oral mucosal surface^28^. Similarly, only the lineage of *Helicobacter pylori* carried *cag*+, *vacA*+, and *babA*+ mutant genes sustained colonization capability in the human stomach, and the inactivation of any of these functions decreases bacterial fitness^29^.

It is clear that the native microbiome, rather than the host, was primarily responsible for probiotic mutations. We elaborated on reasons that can explain this observation. (i) Probiotics may have far fewer interactions with host immunity than pathogens widely documented for surviving in the gut luminal environment^27^. Notably, our evidence in this study showed that probiotics can have a large population size in germ-free mice, yet only <15 genetic mutations occurred, suggesting that a minimal selection pressure derived from hosts. (ii) Probiotics have intrinsic ecological links with native gut microbiota. They are often derived from microbial species (e.g., BV9 in this study) that are naturally adapted to the gut environment or may have evolved mechanisms to rapidly adapt to gut microbiota changes. This could include high mutation rates, as well as other mechanisms such as gene transfer, recombination, and regulation of gene expression^30,31^. (iii) It is generally accepted that the nutrients available in the gut can be limited and may not be sufficient to support the growth and metabolism of both endogenous gut microbes and exogenous probiotics. Thus, the limited access to resources often greatly intensified the microbial competition and evolution/mutation rate of both exogenous probiotics and native microbiota. Resource competition is often implicated as the mechanism driving diversification in this system. Collectively, we disentangled the host and microbiome contribution to in vivo probiotic genetic changes and laid the theoretical foundation for genetically engineering probiotics through in vitro artificial gut ecosystems for their better engraftment and performance in humans.

## Methods

### The study subjects and experimental design

Two model probiotic strains *Lactiplantibacillus plantarum* HNU082 and *Bifidobacterium animalis* subsp. *lactis* V9 (BV9) were used in this animal experiment. The 12 male SPF mice (C57BL/6, 10 weeks age, Shanghai Slack Experimental Animal Co., Ltd., China) and 6 male GF mice (C3H, 10 weeks age, Germ-free C3H mice Laboratory Animal Center, Jiangnan University) were used as the experimental models. All mice were kept on a 12-h light/12-h dark cycle with single cage feeding. The feeding temperature was 25 ± 2 °C, and the humidity was 55% ± 5%. For SPF mice, autoclaved water and irradiated breeding feed were free to access; for GF mice, water and rodent feed were all needed to autoclave. Besides, mattresses, sheds, nests, water, and feed for GF mice were replaced every two days. After being adapted for seven days, the mice were employed to conduct the experiments. The Ethics Committee of Hainan University, China, approved the experimental animal protocols (No. HNUAUCC-2021-00041).

We administered the Lp082 (10^8^ cfu/day) respectively to 3 GF mice and 6 SPF mice for seven days, and BV9 (10^8^ cfu/day) was also respectively administered to 3 GF mice and 6 SPF mice for seven days. Then the isolates were obtained from feces on the following 1, 3, 5, and 7 days (Fig. 1a). Obtaining single bacterial isolates isolated from feces was considered the endpoint of this research. Then, the mice were subjected to intraperitoneal injection of 1% pentobarbital sodium and subsequently humanely euthanized through cervical dislocation following the ARRIVE reporting guidelines^32^. The isolation and taxonomic identification of Lp082 from mice feces were conducted according to our previous studies^7^. BV9 was isolated from the culture medium by mixing the diluted stool samples of mice and 50 mg/L mupirocin, and single colonies were identified by PCR using strain-specific DNA primers (forward: 5’-CGCATATCGCCAACCAAG-3’; reverse: 5’-ACTGCCGAGCGTAAAGCC-3’). In the SPF mice experiment, a total of 51 isolates of Lp082 were subjected to sequencing analysis, while 10 isolates of BV9 were sequencing. In the GF mice experiment, 12 fecal samples from Lp082 and 12 fecal samples from BV9 were sequenced for reference genome mutation annotation.

### The SNV calling of gut-adapted probiotic mutants

After these probiotic isolates were whole-genome re-sequenced, sequencing reads were mapped against the reference genome of the original strains to obtain SNVs using inStrain v1.0.0 (https://github.com/MrOlm/inStrain). The concrete parameters include inStrain profile *.sorted.bam *.fa -c 100 -f 0.49 -o *.profile -p -g ref_genes.fna^33^. In particular, inStrain calculates the ANI between all read pairs and the genomes they map to. Any sequencing reads in a sample with the read-pair ANI to the reference genome <95% were, by default, abandoned. A sample (i.e., isolate genomic sequence) with a mean read ANI <0.95 were discarded. Meanwhile, the genomic functions of each strain were annotated by Prodigal^34^.

### Fecal shotgun metagenomic sequencing and microbial species profiling

To ensure and confirm that the original probiotic strains were not present in the mice’s resident gut tract before their entry and to track the microbial population dynamics of gut microbiota after probiotic consumption, we collected the feces before and after administering the probiotics and conducted shotgun metagenomic sequencing to identify and estimate the abundance of gut inhabitants including ingested probiotics. The QIAamp® DNA Stool Mini Kit (Qiagen, Hilden, Germany) was used for metagenomic DNA extraction. The purity and integrity of DNA were evaluated by 0.8% agarose gel electrophoresis. Specifically, the purity of DNA was detected based on the ratio of OD 260/280 by Nanodrop^35^, and the concentration of DNA was accurately quantified by Qubit® DNA Assay Kit in Qubit® 3.0 Fluorometer (Invitrogen, USA)^36^. All DNA samples were sequenced by Illumina HiSeq 2500 instrument in the Novogene Company (Beijing, China). With fecal shotgun metagenomics sequencing, we further predicted the relative abundance of gut resident species and probiotic strains using MetaPhlAn3^37^.

### Reporting summary

Further information on research design is available in the Nature Research Reporting Summary linked to this article.

### Supplementary information

Supplemental table

Reporting summary

## Data Availability

The datasets generated during the current study are available in the NCBI repository, with the accession number: PRJNA933377.
