# Supplementary material for: Native microbiome dominates over host factors in shaping the probiotic genetic evolution in the gut
Source: NPJ Biofilms Microbiomes. 2023 Oct 14;9:80. doi: 10.1038/s41522-023-00447-8 (PMC10576824; doi:10.1038/s41522-023-00447-8)
Supplement: Supplementary file 2 — Reporting summary [file 41522_2023_447_MOESM2_ESM.pdf]

nature portfolio

Corresponding author(s): Jiachao Zhang

Last updated by author(s): Sep 1, 2023

Reporting Summary

Nature Portfolio wishes to improve the reproducibility of the work that we publish. This form provides structure for consistency and transparency in reporting. For further information on Nature Portfolio policies, see our Editorial Policies and the Editorial Policy Checklist.

Statistics

For all statistical analyses, confirm that the following items are present in the figure legend, table legend, main text, or Methods section.

n/a	Confirmed	
☐	☒	The exact sample size (*n*) for each experimental group/condition, given as a discrete number and unit of measurement
☐	☒	A statement on whether measurements were taken from distinct samples or whether the same sample was measured repeatedly
☐	☒	The statistical test(s) used AND whether they are one- or two-sided<br>*Only common tests should be described solely by name; describe more complex techniques in the Methods section.*
☒	☐	A description of all covariates tested
☒	☐	A description of any assumptions or corrections, such as tests of normality and adjustment for multiple comparisons
☒	☐	A full description of the statistical parameters including central tendency (e.g. means) or other basic estimates (e.g. regression coefficient) AND variation (e.g. standard deviation) or associated estimates of uncertainty (e.g. confidence intervals)
☒	☐	For null hypothesis testing, the test statistic (e.g. *F*, *t*, *r*) with confidence intervals, effect sizes, degrees of freedom and *P* value noted<br>*Give P values as exact values whenever suitable.*
☒	☐	For Bayesian analysis, information on the choice of priors and Markov chain Monte Carlo settings
☒	☐	For hierarchical and complex designs, identification of the appropriate level for tests and full reporting of outcomes
☒	☐	Estimates of effect sizes (e.g. Cohen's *d*, Pearson's *r*), indicating how they were calculated

Our web collection on statistics for biologists contains articles on many of the points above.

Software and code

Policy information about availability of computer code

Data collection	The Ethics Committee of Hainan University, China, approved the experimental animal protocols (No. HNUAUCC-2021-00041). We administered the Lp082 (108cfu) respectively to 3 GF mice and 6 SPF mice for seven days, and BV9 (108cfu) was also respectively administered to 3 GF mice and 6 SPF mice for seven days. Then the isolates were obtained from feces on the following 1, 3, 5, and 7 days.In the SPF mice experiment, a total of 51 isolates of Lp082 were subjected to sequencing analysis, while 10 isolates of BV9 were sequencing. In the GF mice experiment, 12 fecal samples from Lp082 and 12 fecal samples from BV9 were sequenced for reference genome mutation annotation. We also collected the feces before and after administering the probiotics and conducted shotgun metagenomic sequencing.
Data analysis	PCoA analysis were used to analyze the time and individual influence. The Spearman's rank correlation analysis between a resident and probiotic strain was carried using a free statistical software R. The SNV calling was obtained using InStrain v1.0.0 (https://github.com/MrOlm/inStrain) with code (inStrain profile *.sorted.bam *.fa -c 100 -f 0.49 -o *.profile -p -g ref_genes.fna).

For manuscripts utilizing custom algorithms or software that are central to the research but not yet described in published literature, software must be made available to editors and reviewers. We strongly encourage code deposition in a community repository (e.g. GitHub). See the Nature Portfolio guidelines for submitting code & software for further information.

Data

Policy information about availability of data

All manuscripts must include a data availability statement. This statement should provide the following information, where applicable:

- Accession codes, unique identifiers, or web links for publicly available datasets
- A description of any restrictions on data availability
- For clinical datasets or third party data, please ensure that the statement adheres to our policy

The datasets generated during the current study are available in the NCBI repository, with the accession numbers: PRJNA933377.

Research involving human participants, their data, or biological material

Policy information about studies with human participants or human data. See also policy information about sex, gender (identity/presentation), and sexual orientation and race, ethnicity and racism.

Reporting on sex and gender	Not applicable
Reporting on race, ethnicity, or other socially relevant groupings	Not applicable
Population characteristics	Not applicable
Recruitment	Not applicable
Ethics oversight	Not applicable

Note that full information on the approval of the study protocol must also be provided in the manuscript.

Field-specific reporting

Please select the one below that is the best fit for your research. If you are not sure, read the appropriate sections before making your selection.

☒ Life sciences ☐ Behavioural & social sciences ☐ Ecological, evolutionary & environmental sciences

For a reference copy of the document with all sections, see nature.com/documents/nr-reporting-summary-flat.pdf

Life sciences study design

All studies must disclose on these points even when the disclosure is negative.

Sample size	The 12 male SPF mice and 6 male GF mice were used as the experimental models. In the SPF mice experiment, a total of 51 isolates of Lp082 were subjected to sequencing analysis, while 10 isolates of BV9 were sequencing. In the GF mice experiment, 12 fecal samples from Lp082 and 12 fecal samples from BV9 were sequenced for reference genome mutation annotation.
Data exclusions	Not applicable
Replication	Not applicable
Randomization	After one week of adaptive feeding, each mouse was assigned a unique identifier. Subsequently, a complete randomization process was employed based on these identifiers.
Blinding	Not applicable

Reporting for specific materials, systems and methods

We require information from authors about some types of materials, experimental systems and methods used in many studies. Here, indicate whether each material, system or method listed is relevant to your study. If you are not sure if a list item applies to your research, read the appropriate section before selecting a response.

Materials & experimental systems

n/a	Involved in the study
☒	☐ Antibodies
☒	☐ Eukaryotic cell lines
☒	☐ Palaeontology and archaeology
☐	☒ Animals and other organisms
☒	☐ Clinical data
☒	☐ Dual use research of concern
☒	☐ Plants

Methods

n/a	Involved in the study
☒	☐ ChIP-seq
☒	☐ Flow cytometry
☒	☐ MRI-based neuroimaging

Animals and other research organisms

Policy information about studies involving animals; ARRIVE guidelines recommended for reporting animal research, and Sex and Gender in Research

Laboratory animals	C57BL/6 and C3H mice (10 weeks age) were employed to conduct experiment.
Wild animals	Not applicable
Reporting on sex	All mice are male.
Field-collected samples	All mice were kept on a 12-hour light/12-hour dark cycle with single cage feeding. The feeding temperature was 25±2°C, and the humidity was 55%±5%. For SPF mice, autoclaved water and irradiated breeding feed were free to access; for GF mice, water and rodent feed were all needed to autoclave. The diluted stool samples of mice were collected and isolated probiotics immediately.
Ethics oversight	The Ethics Committee of Hainan University, China, approved the experimental animal protocols (No. HNUAUCC-2021-00041).

Note that full information on the approval of the study protocol must also be provided in the manuscript.
